# Socioeconomic and sociodemographic correlations to COVID-19 variability in the United States in 2020

**DOI:** 10.3389/fpubh.2024.1359192

**Published:** 2024-06-11

**Authors:** Nikolay Golosov, Shujie Wang, Manzhu Yu, Nakul N. Karle, Oye Ideki, Bishara Abdul-Hamid, Christopher Blaszczak-Boxe

**Affiliations:** ^1^Department of Geography, The Pennsylvania State University, University Park, PA, United States; ^2^Earth and Environmental Systems Institute, The Pennsylvania State University, University Park, PA, United States; ^3^Department of Earth, Environment and Equity, Howard University, Washington, DC, United States; ^4^Department of Learning, Design, and Technology, Department of Performing Systems, College of Education, The Pennsylvania State University, University Park, PA, United States

**Keywords:** COVID-19, PM_2.5_, socioeconomic variables, ordinary least squares (OLS), random forest (RF), statistical correlation

## Abstract

The COVID-19 pandemic provided an additional spotlight on the longstanding socioeconomic/health impacts of redlining and has added to the myriad of environmental justice issues, which has caused significant loss of life, health, and productive work. The Centers for Disease Control and Prevention (CDC) reports that a person with any selected underlying health conditions is more likely to experience severe COVID-19 symptoms, with more than 81% of COVID-19-related deaths among people aged 65 years and older. The effects of COVID-19 are not homogeneous across populations, varying by socioeconomic status, PM_2.5_ exposure, and geographic location. This variability is supported by analysis of existing data as a function of the number of cases and deaths per capita/1,00,000 persons. We investigate the degree of correlation between these parameters, excluding health conditions and age. We found that socioeconomic variables alone contribute to ~40% of COVID-19 variability, while socioeconomic parameters, combined with political affiliation, geographic location, and PM_2.5_ exposure levels, can explain ~60% of COVID-19 variability per capita when using an OLS regression model; socioeconomic factors contribute ~28% to COVID-19-related deaths. Using spatial coordinates in a Random Forest (RF) regressor model significantly improves prediction accuracy by ~120%. Data visualization products reinforce the fact that the number of COVID-19 deaths represents 1% of COVID-19 cases in the US and globally. A larger number of democratic voters, larger per-capita income, and age >65 years is negatively correlated (associated with a decrease) with the number of COVID cases per capita. Several distinct regions of negative and positive correlations are apparent, which are dominated by two major regions of anticorrelation: (1) the West Coast, which exhibits high PM_2.5_ concentrations and fewer COVID-19 cases; and (2) the middle portion of the US, showing mostly high number of COVID-19 cases and low PM_2.5_ concentrations. This paper underscores the importance of exercising caution and prudence when making definitive causal statements about the contribution of air quality constituents (such as PM_2.5_) and socioeconomic factors to COVID-19 mortality rates. It also highlights the importance of implementing better health/lifestyle practices and examines the impact of COVID-19 on vulnerable populations, particularly regarding preexisting health conditions and age. Although PM_2.5_ contributes comparable deaths (~7M) per year, globally as smoking cigarettes (~8.5M), quantifying any causal contribution toward COVID-19 is non-trivial, given the primary causes of COVID-19 death and confounding factors. This becomes more complicated as air pollution was reduced significantly during the lockdowns, especially during 2020. This statistical analysis provides a modular framework, that can be further expanded with the context of multilevel analysis (MLA). This study highlights the need to address socioeconomic and environmental disparities to better prepare for future pandemics. By understanding how factors such as socioeconomic status, political affiliation, geographic location, and PM_2.5_ exposure contribute to the variability in COVID-19 outcomes, policymakers and public health officials can develop targeted strategies to protect vulnerable populations. Implementing improved health and lifestyle practices and mitigating environmental hazards will be essential in reducing the impact of future public health crises on marginalized communities. These insights can guide the development of more resilient and equitable health systems capable of responding effectively to similar future scenarios.

## 1 Introduction

Environmental justice is the fair treatment and meaningful engagement of all people, regardless of ethnic group, color, national origin, or income, to the development and enforcement of environmental laws, regulations, and policies, ensuring that no community disproportionately bears the burden of environmental hazards while also promoting access to environmental benefits (1–6). The legacy of redlining has exacerbated socioeconomic and demographic inequalities and public health disparities, which are further intensified by climate change and escalate air pollution; this has negative consequences on human health globally, contributing to a significant portion of annual deaths, disproportionately affecting impoverished nations, and resulting in widespread environmental and economic challenges. Particulate matter (PM), a significant component of air pollution resulting from various natural and anthropogenic activities, is a major contributor to adverse human health impacts. Suspended in the atmosphere over extended periods and capable of traversing long distances, PM becomes a pervasive concern with far-reaching consequences for public health (7). Pollution, overall (when including water, occupational, and lead contamination), contributes to ~9 M deaths/year, which is comparable to smoking/secondhand smoking-related deaths (8).

**Figure 1:**
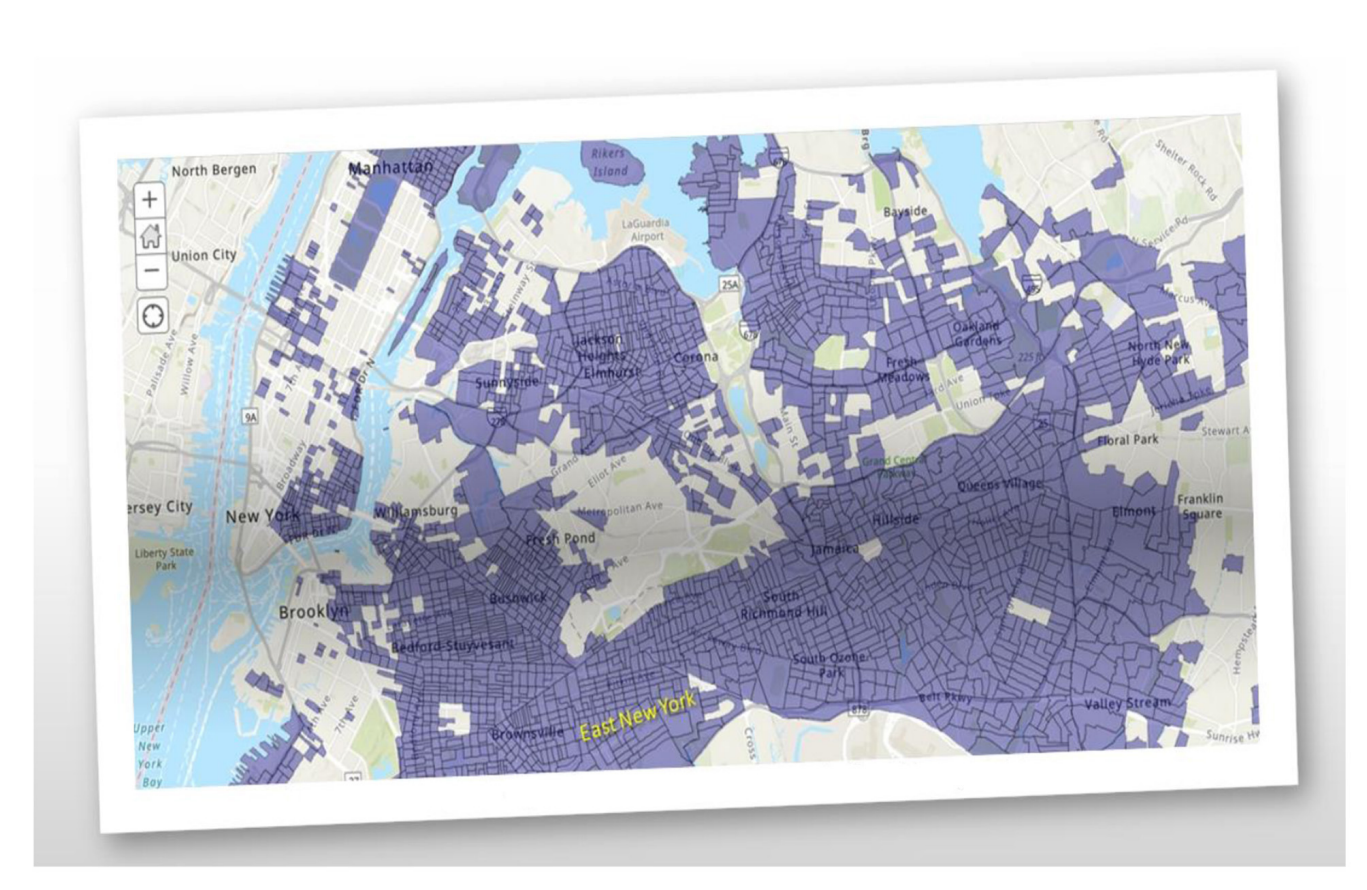
Environmental justice areas in New York City.

Health disparities are key indicators of how overall health and economics are so intertwined. The ability to live a long and healthy life is not equally available to all. A baby born to a family that lives in the Upper East Side will live 7.5–11 years longer than a baby born to a family in East New York/Starrett City, Bedford-Stuyvesant, and Brownsville, Brooklyn (9, 10). As of 2016, the median household income of East New York residents was $36,780, exhibited a 10% and 30% unemployment and poverty rate, respectively, and have a 52% rent burden. Cancer, heart, and respiratory disease are the leading causes of premature death, like the rest of NYC (11, 12). LI neighborhoods and BIPOC New Yorkers are dying before 65 at higher rates. East New York & Starrett City, Brooklyn exhibited one of the highest COVID-19 mortality rates in the world during the Spring 2020 phase of the pandemic. Figure 1 exemplifies EJ-designated areas throughout NYC via EPA's ArcGIS - My Map + EJScreen.

Considering the persistent COVID-19 pandemic, which has resulted in over 104 million confirmed cases and 1.125 million fatalities in the United States, understanding the intricate connections between socioeconomic factors, air pollution, and the variability in COVID-19 outcomes is crucial (13, 14). Understanding the link between air pollution and COVID-19 outcomes is crucial because prior research suggests that exposure to pollutants like PM_2.5_ and PM_10_ can suppress immune responses, amplify inflammation, and damage cells, potentially intensifying COVID-19 severity (15), especially in already vulnerable populations (16–23). These findings are further underscored by studies that indicate a significant association between increased PM_2.5_ levels and elevated COVID-19 death rates. Wu et al. (16) found that an increase of only 1 μg/m3 in PM_2.5_ is associated with an 8% increase in the COVID-19 death rate (95% confidence interval [CI]: 2%, 15%). A study conducted in England explored potential links between major fossil fuel-related air pollutants and COVID-19 mortality. The study revealed a positive correlation between pollutant concentrations, particularly nitrogen oxides, and COVID-19 mortality and infectivity (24). In addition, Pozzer et al. (17) estimated that particulate air pollution contributed ~15% (95% confidence interval 7–33%) to COVID-19 mortality worldwide, 27% (13–46%) in East Asia, 19% (8–41%) in Europe, and 17% (6–39%) in North America. Globally, ~50–60% of the attributable anthropogenic fraction is related to fossil fuel use, up to 70–80% in Europe, West Asia, and North America. Additionally, socioeconomic disparities, such as poverty and lack of healthcare access, which are intertwined with higher pollution exposure, play a pivotal role in the spread and impact of the virus. Thus, examining the interplay between socioeconomic factors, air pollution, and COVID-19 can shed light on targeted interventions to mitigate disease impact in high-risk communities. Within this context, accurate modeling, forecasting, and mitigation strategies are hard and non-trivial to implement when aiming to quantitatively understand COVID-19 mortality and morbidity correlations to socioeconomic and sociodemographic confounding factors. Using transmission and exposure models, recent studies provide high degrees of accuracy in forecasting COVID-19 transmission and exposure risks (25–29); in addition, these studies show that international trade serves as a surrogate indicator reflecting human-to-human interactions, encompassing various drivers influencing the transmission dynamics of COVID-19 across geoeconomic regions. It is further regarded as a proxy for social parameters applicable to local communities, albeit challenging to model accurately. Contrary to merely representing economic exchanges, international trade reflects intricate social dynamics. Research suggests that regions with heightened trade activities exhibit higher confirmed cases of COVID-19 infection, attributable to increased social interactions accompanying economic expansion. The correlation between infection rates and international trade is closely tied to GDP, indicating elevated interpersonal interactions as economic activities surge. This parameter demonstrates a linear relationship with the virus spread across all regions of the USA. Furthermore, it correlates with COVID-19 outcomes. Additional studies, using IoT-enabled platforms have created automated techniques to further support the reduction in transmission of COVID-19 by way of novel hand sanitizers (30, 31). More targeted approaches have also been developed to better mitigate air quality emissions through data analytics and scientometric analysis (32).

This study examines the impact of socioeconomic variables, voting outcomes, and air pollution (i.e., PM_2.5_) on the temporal variability/number of COVID-19 cases and deaths in the US. The study uses data from the CDC and the US Census Bureau, and ~ 5000 ambient PurpleAir PM_2.5_ sensors. Data on socioeconomic and sociodemographic variables, voting outcomes, air pollution, and the number of COVID-19 cases and deaths for the lower 48 states was obtained from the CDC and the US Census Bureau, and ~5,000 ambient PurpleAir PM_2.5_ sensors, which were all merged to construct the data analytics products are described here.

Specific questions addressed in this study are as follows:

- How significantly do factors like socioeconomic variables, political affiliation, and air pollution (specifically, PM_2.5_) contribute to the number of COVID-19 cases and deaths?
- Are there any identifiable geographic patterns on the spread and impact of COVID-19?
- Is there a significant relationship between PM_2.5_ air pollution and COVID-19 outcomes?
- How can increased cross-/interdisciplinary research in core STEM disciplines with the Education research realm, contribute to the increased representation of Black, Brown, and low-income populations in the workforce—especially within the context that educational attainment is the primary governing factor that positively correlates with increased economic mobility and potential? (33, 34).

## 2 Experimental design and methods

The study used sociodemographic variables, voting outcomes, COVID-19 cases, death numbers, and air pollution data—all obtained from the CDC and the US Census Bureau, and ~5000 ambient PurpleAir PM_2.5_ sensors. Table 1 provides a summary of the variables used in this study. The Minority Social Vulnerability (SVI) (35) index provided a vetted set of sociodemographic variables from the US Census American Community Survey (ACS) that the CDC determined most influential in COVID-19 spread. We used data from both the standard ACS and a curated list from the CDC's Minority SVI. Notably, the Minority SVI differs by including four extra variables about specific minority groups (Spanish, Chinese, Vietnamese, and Russian speakers and their fluency).

**Table 1:** List of the data sources and their descriptions.

**Data source name**	**Variables**	**Description**	**URL**
Purpleair	PM_2.5_ outdoor air pollution data	PurpleAir makes sensors that a community of citizen scientists uses to collect hyper-local, real-time air quality data and share it on a map that is accessible to everyone.	purpleair.com web map
Minority SVI	Sociodemographic variables, selected by CDC as influential for pandemic spread	Minority Health SVI uses data from the United States Census Bureau and other public sources to help identify communities that may need support before, during, and after disasters, with a focus on minority racial, ethnic, and language groups as well as medical vulnerability.	CDC Onemap
County Presidential Election Returns 2000-2020	% voted for Democrat, Republican, Green, Libertarian	This dataset contains county-level returns for presidential elections from 2000 to 2020 (2021-06-08).	Harvard Dataverse
Time series summary (csse_covid_19_time_series)	COVID-19 cases and deaths on 12/31/2020	Data from JHU. This data contains daily time series summary tables: confirmed, deaths, and recovered. All data is read from the daily case report.	GITHUB

We obtained our COVID-19 case and death statistics from the Johns Hopkins University database. The data we selected, as of 12/31/2020, was standardized per 1,00,000 people. This date was chosen to align with a future CDC death certificate dataset from 2020, as CDC datasets are released a year later (36). For political data, we used MIT's database on county presidential election outcomes. However, the data for regions like Hawaii, Alaska, Guam, and Puerto Rico differed, as they were grouped by voting districts, not counties, preventing a straightforward integration with county-level data.

Regarding the air quality data, there are two main data sources for the US—a federal website AirNow.gov [governed by the Environmental Protection Agency (EPA)] and PurpleAir.com (a private company). EPA's AirNow.gov uses calibrated sensors and provides high-quality data for a limited number of locations, across the US, whereas, the PurpleAir provides more sensor coverage/locations, at the cost of lower accuracy and precision (37). We downloaded daily air pollution data (daily averages) for all available sensors within the 48 lower states between 2/1/20 and 12/31/20. To ensure acceptable data quality, sensors with less than 100 days of observations and average PM_2.5_ values equal to zero or >500. There were 3,344 sensors selected in 574 counties (out of 3,071 counties and county equivalents). The average values of PM_2.5_ for each sensor for the said period were calculated.

The first three data sources provided tabular data summarized by US counties or county equivalents with associated Federal Information Processing Standard (FIPS) codes. FIPS codes are an unambiguous numeric identifier of administrative units in the US (38). Having this identifier in the source data streamlined the process of joining these data tables with each other. To further look at the spatial relationships in the data, we synthesized the resulting non-spatial table with sociodemographic variables with the spatial layer containing US county boundaries.

As a result of the data preparation process, the master Comma-Separated Value (CSV) file was created, containing all counties within the lower 48 states with associated sociodemographic variables, voting outcomes and number of COVID-19 cases/deaths (per capita of 100K persons), and associated interpolated PM_2.5_ pollution counts, extracted from the IDW raster created in ArcGIS Spatial Analyst (more in the Methods section). The list of the data sources is provided in Table 1, and the full list of variables in the resulting dataset is provided in Table 2.

**Table 2:** List of variables.

**Name of the variable**	**Description**	**Units**	**Source**
R_CASES	COVID cases per 1,00,000 on 12/31/2020	Ratio per 1,00,000	JHU
R_DEATHS	COVID deaths per 1,00,000 on 12/31/2020	Ratio per 1,00,000	JHU
EP_POV	Population below the poverty level	Percentage	CDC MSVI
EP_UNEMP	Unemployed	Percentage	CDC MSVI
EP_PCI	Per capita income	Percentage	CDC MSVI
EP_AGE65	Population on or above 65 years old	Percentage	CDC MSVI
EP_AGE17	Population on or below 17 years old	Percentage	CDC MSVI
EP_DISABL	Disabled population	Percentage	CDC MSVI
EP_SNGPNT	Single parent	Percentage	CDC MSVI
EP_AIAN	Native American/Alaskan Native	Percentage	CDC MSVI
EP_AFAM	African American	Percentage	CDC MSVI
EP_NHPI	Native Hawaian/Pacific islander	Percentage	CDC MSVI
EP_HISP	Hispanic population	Percentage	CDC MSVI
EP_SPAN	Speakers who speak English less than “Very well” and is a Spanish speaker	Percentage	CDC MSVI
EP_CHIN	-“- Chinese speaker	Percentage	CDC MSVI
EP_VIET	-“- Vietnamese speaker	Percentage	CDC MSVI
EP_KOR	-“- Korean speaker	Percentage	CDC MSVI
EP_RUS	-“- Russian speaker	Percentage	CDC MSVI
EP_MUNIT	Multiple unit housing	Percentage	CDC MSVI
EP_MOBILE	Mobile homes	Percentage	CDC MSVI
EP_CROWD	Crowdedness (percentage of households where a number of people > number of rooms)	Percentage	CDC MSVI
EP_NOVEH	Number vehicle access	Percentage	CDC MSVI
EP_GROUPQ	Population in group quarters (oilfield camps, army camps, prisons, nursing homes)	Percentage	CDC MSVI
R_HOSP	Hospitals per 1,00,000	Ratio per 1,00,000	CDC MSVI
R_URG	Urgent care units per 1,00,000	Ratio per 1,00,000	CDC MSVI
R_PHARM	Pharmacies per 1,00,000	Ratio per 1,00,000	CDC MSVI
R_PCP	Primary care providers per 1,00,000	Ratio per 1,00,000	CDC MSVI
EP_UNINSUR	Uninsured	Percentage	CDC MSVI
EP_NOINT	No internet access	Percentage	CDC MSVI
EP_NOHSDP	No high-speed internet access	Percentage	CDC MSVI
EP_DEMOCRAT	Voted for Democratic presidential candidate	Percentage	MIT
EP_REPUBLICAN	-“- Republican	Percentage	MIT
EP_LIBERTARIAN	-“- Libertarian	Percentage	MIT
EP_GREEN	-“- Green	Percentage	MIT
EP_OTHER	-“- Other	Percentage	MIT

Variables names start with R (Ratio per 100 000) and EP (Estimated %).

To understand to which degree there is a relationship between PurpleAir PM_2.5_ data, selected sociodemographic parameters, voting outcomes, and COVID-19 cases and deaths, an ordinary least squares regression was employed (39), using cases/deaths per 1,00,000 persons as dependent variables and all other variables as independent variables.

To assess the possible influence of certain independent variables on the dependent variable, scatter plots of the variable in question vs. the independent variable are plotted as well as Pearson correlation coefficients and *p*-values are calculated to assess the correlation strength. To assess the possible multicollinearity issues, a correlation plot (heatmap) was employed.

Next, the Variance Inflation Factor (VIF) coefficients were calculated, showing the degree of multicollinearity. VIFs range between 1 and 0 multicollinearity to infinity. The common rule of thumb is that if the VIF for a specific variable is >five, these variables exhibit strong multicollinearity. If one is interested in interpretable regression coefficients, then remove or incorporate the variable of focus with other variables (40).

To facilitate the interpretation of the regression results, the data was preprocessed using sklearn Standard Scaler (standardized and scaled data by subtracting the mean and scaling to the unit variance). Outliers were removed using a Local Outlier Factor. These steps are useful to further compare the regression coefficients that allowed us to see the more influential variables. The Statsmodels.api.ols Python module as well as ArcGIS Ordinary Least Squares (OLS) tool were used to run the regression analysis. These tools output the same regression coefficients and R2 metrics, although the ArcGIS OLS tool provides an easy way to output the residuals into the source dataset. It is useful if one desires to explore how residuals are geographically distributed.

Regression results were checked for multicollinearity in Regression Analysis and regression assumptions using the Residuals vs. Fit graph and Q–Q residual plotting. The assumption is that the regression residuals should be independent and normally distributed, so the model can be used for inference (41). Otherwise, it could be useful for prediction but useless for interpretation. The overall predictive accuracy of the model was assessed using its R2 scores on a randomly selected unseen test dataset (25% of the data) and using five-fold cross-validation.

### 2.1 Influence of location/distinctive spatial patterns of COVID-19 development

Moran I test on residuals was employed to check if the residuals exhibit spatial autocorrelation (42). This test is significant when the data exhibits clustering or anticlustering patterns and is insignificant if the spatial distribution is random (43). Having confirmed the non-random clustered distribution of residuals, one can conclude that either some of the important explanatory variables were missed from the model or there is an influence of some spatial process so the model systematically over/underpredicts the dependent variable at specific places. A random distribution (a non-significant Moran's I) signals a well-fitting model, but, it raises the alarm when the test flags a significant pattern. Two possibilities emerge: either vital explanatory variables are missing, leaving spatial factors to unfairly influence the dependent variable at specific locations, or hidden spatial processes, interactions between existing variables are distorting the model's predictions.

To test if the added spatial coordinate features help to predict the dependent variable more accurately, the random forest regressor from the SKLearn was used. The spatial coordinates of centroids of each US county were extracted into separate X and Y variables and further used as the independent variables in a random forest regressor. The Random Forest Regressor is a machine learning algorithm that constructs multiple decision trees using various sub-samples of a dataset and aggregates the individual decision tree results through averaging to improve the prediction accuracy (44). The algorithm was run with recommended parameters of max-features set to None and max-depth set to None. The first parameter controls how many features to consider making a split in the decision tree, and all features are included when set to None. Max-depth controls the size of the individual tree; the N-estimator parameter, which controls the number of trees in the forest, was set to 500 (the default value is 100). It helps to achieve a bit better accuracy by the price of consuming more computing resources.

To assess the predictive accuracy of the Random Forest regression model, similar to OLS regression, a test dataset R2 score and k-fold cross-validation techniques were employed. To assess the relative importance of different predictors in the model, Permutation Importance accesses a decrease in a model predictive score if one of the predictive variables is randomly shuffled. The bigger the drop in the score the more the predictor is important (45).

### 2.2 Influence of PM_2.5_ pollutant concentrations on the numbers of COVID-19 cases/deaths

Given the limited coverage of PurpleAir sensors, encompassing only one-sixth of US counties, spatial interpolation was employed to estimate average PM_2.5_ values in non-monitored areas. Recognizing the decreasing influence of distant readings, we utilized the Inverse Distance Weighting (IDW) method using ArcGIS (46). This technique generates a spatially continuous raster dataset of PM_2.5_ concentrations across the entire US landmass. To facilitate county-level analysis, the raster data was then converted back into point data, allowing us to extract average PM_2.5_ values for each county. By employing this approach, we were able to overcome the data sparsity issue and create a comprehensive picture of PM_2.5_ distribution across the entire United States.

## 3 Results and discussion

**Figure 2:**
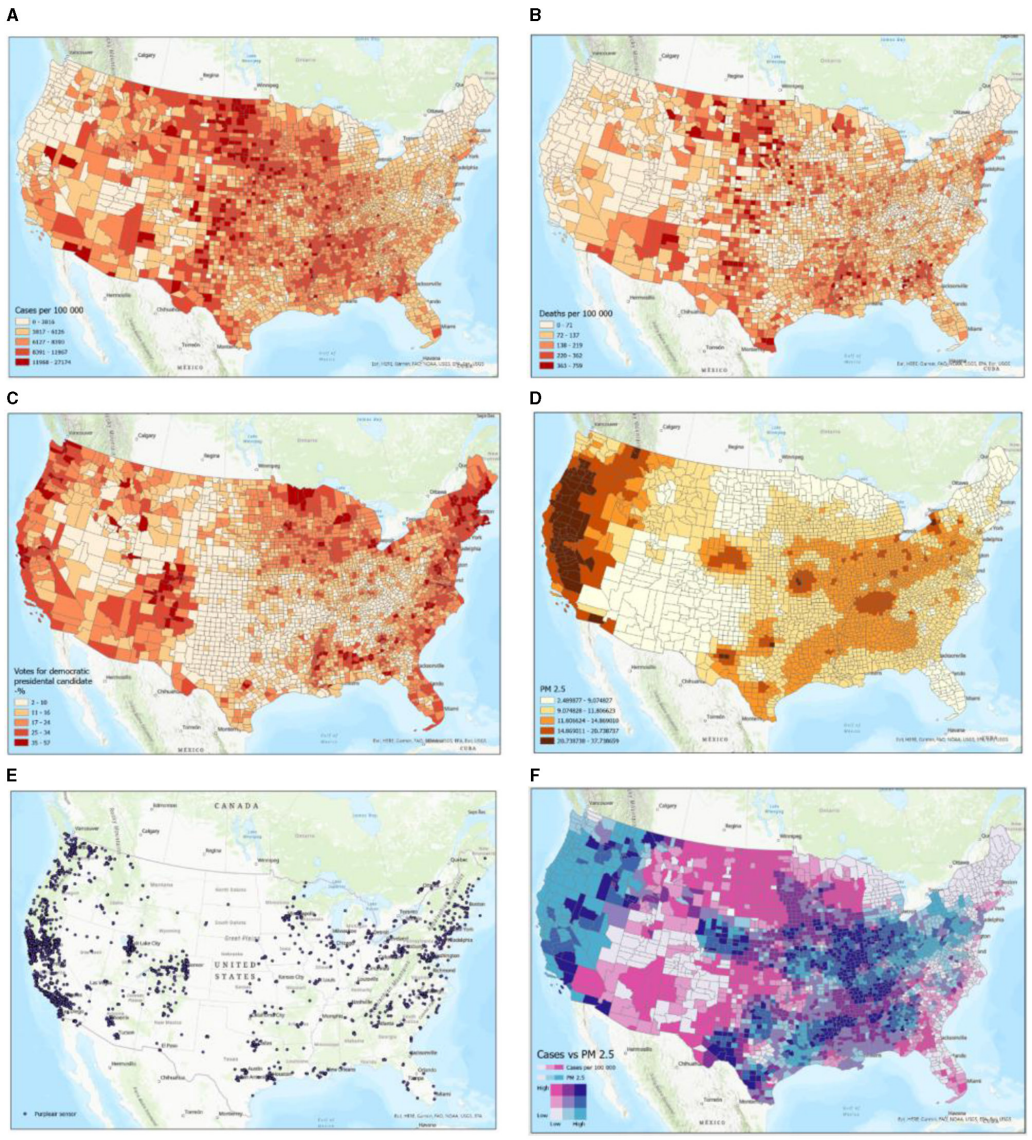
Examples of participating datasets in map format. Schemes follow another format. If there are multiple panels, they should be listed as **(A)** COVID-19 cases per 1,00,000 persons; **(B)** COVID-19 deaths per 1,00,000 persons; **(C)** Voting outcomes (% Democrats); **(D)** Average PM_2.5_ air pollution (IDW interpolation); **(E)** Purpleair sensor locations; and **(F)** PM_2.5_ exposure levels vs. COVID-19 cases per 1,00,000 persons.

Qualitatively, Figures 2A–F do not exhibit any positive or negative correlations among all of them. Between specific subset figures of Figure 2, general statements can be made to describe their relationships. For example, Figures 2A, B exhibit positive correlations, generally, amongst of the eastern portion of the United States, with high positive correlations at the nexus of Nevada, New Mexico, Colorado, and Utah, and the Northern middle states. No specific correlations, especially of high spatial resolution, can be made between these two figures. Yet, Figures 2A, B reinforce the fact that the global and US ratios of deaths/number of COVID-19 cases are 0.01 and 0.011, respectively. In other words, COVID-19-caused deaths represent ~1% of the total number of cases both in the US and globally (https://coronavirus.jhu.edu/). Figure 2C, the percentage of democratic voters, only exhibited general positive correlations with the location of PurpleAir sensors (Figure 2E) and did not show any correlations with any of the other subset figures. Figures 2A, D, F show several distinct regions: (1) the western portion of the US, which exhibits high PM_2.5_ concentrations and low COVID-19 cases; (2) the west-to-middle portion of the US, exhibiting high COVID-19 cases and low PM_2.5_ concentrations; (3) a regional part of the southwestern part of the US, exhibiting low PM_2.5_ concentrations and low COVID-19 cases; (4) an approximately evenly distributed blend of high PM_2.5_ concentrations and high COVID-19 cases, mid-to-high number of COVID-19 cases and low PM_2.5_ concentrations, and high PM_2.5_ concentrations and low number of COVID-19 cases in the middle-to-eastern part of the US; and (5) low PM_2.5_ concentrations and low number of COVID-19 cases in the Northeastern area of the US.

Having inspected the correlation coefficients on the correlation plot, one can conclude that many pairs of variables are strongly correlated. Indeed, Tables 3, 4 show the top five strongly positively and negatively, correspondingly, correlated pairs of variables:

**Table 3:** Top five positive correlations.

**First variable**	**Second variable**	**Correlation coefficient**
EP_SPAN	EP_HISP	0.89
EP_CHIN	EP_ASIAN	0.68
EP_NOINT	EP_POV	0.66
EP_NOINT	EP_NOHSDP	0.65
EP_UNEMP	EP_POV	0.64

**Table 4:** Top five negative correlations.

**First variable**	**Second variable**	**Correlation coefficient**
EP_PCI	EP_POV	−0.72
EP_NOINT	EP_PCI	−0.71
EP_NOHSDP	EP_PCI	−0.64
EP_DISABL	EP_PCI	−0.58
EP_AGE17	EP_AGE65	−0.57

**Figure 3:**
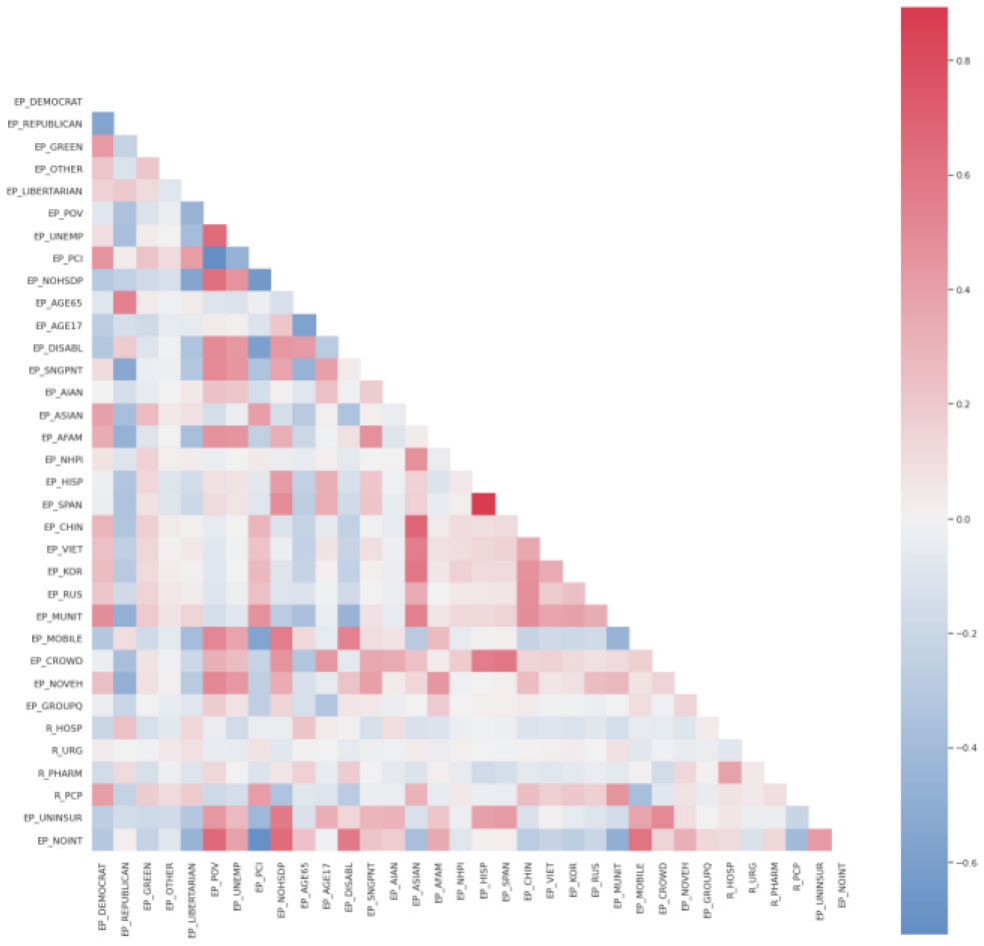
Correlation strength between different variables.

On the other hand, the correlations between the dependent variables and any independent variables are very mild, whereas many independent variables are strongly correlated, which could indicate possible multicollinearity issues. These multicollinearity issues are even more prominent when inspecting Figure 3.

**Figure 4:**
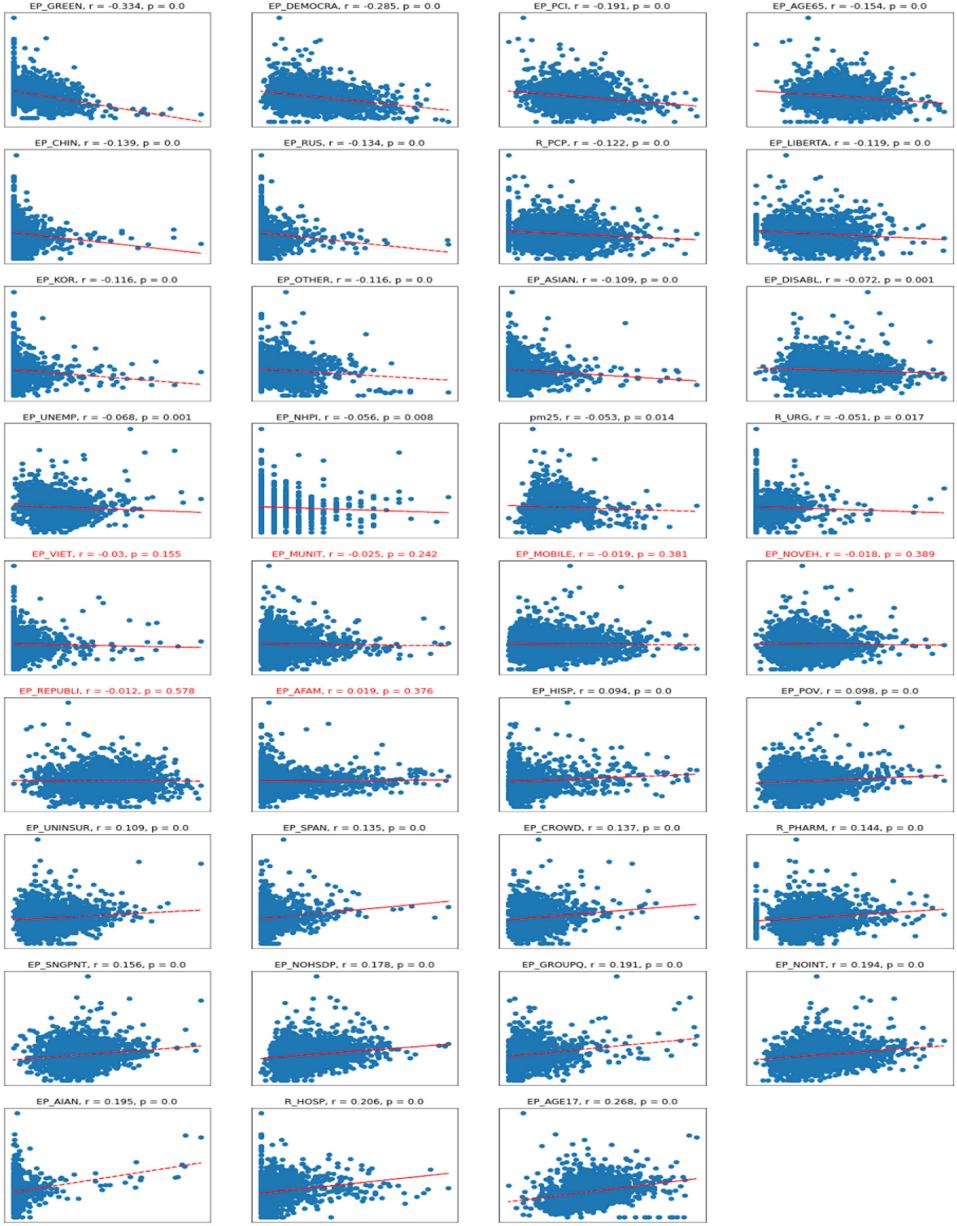
Scatterplots for Cases vs. independent variables. *R*, Pearson correlation; *p*, significance. Titles in red indicate non-significant correlations.

The scatter plots depict the relationships between the dependent variable of cases per 1,00,000 persons and independent variables. As one can see in the correlation plots (Figure 4), the strongest negatively correlated independent variables are various types of political outcomes—percent votes for democrats and greens, as well as per capita income. A larger number of democratic voters, larger per-capita income, and age greater than 65—all these factors are negatively correlated (associated with a decrease) of the number of COVID cases per 1,00,000 persons.

On the other hand, the number of Republican voters has a non-significant and almost zero correlation with the number of cases. Being of young age (younger than 17), staying in group quarters (prisons, nursing homes, oilfield worker/construction camps), having no internet access, and being American Indian or Alaska Native are the factors associated with an increase in COVID-19 cases per capita. There are also some casual correlations between a high number of hospitals per capita associated with a high number of COVID-19 cases.

**Figure 5:**
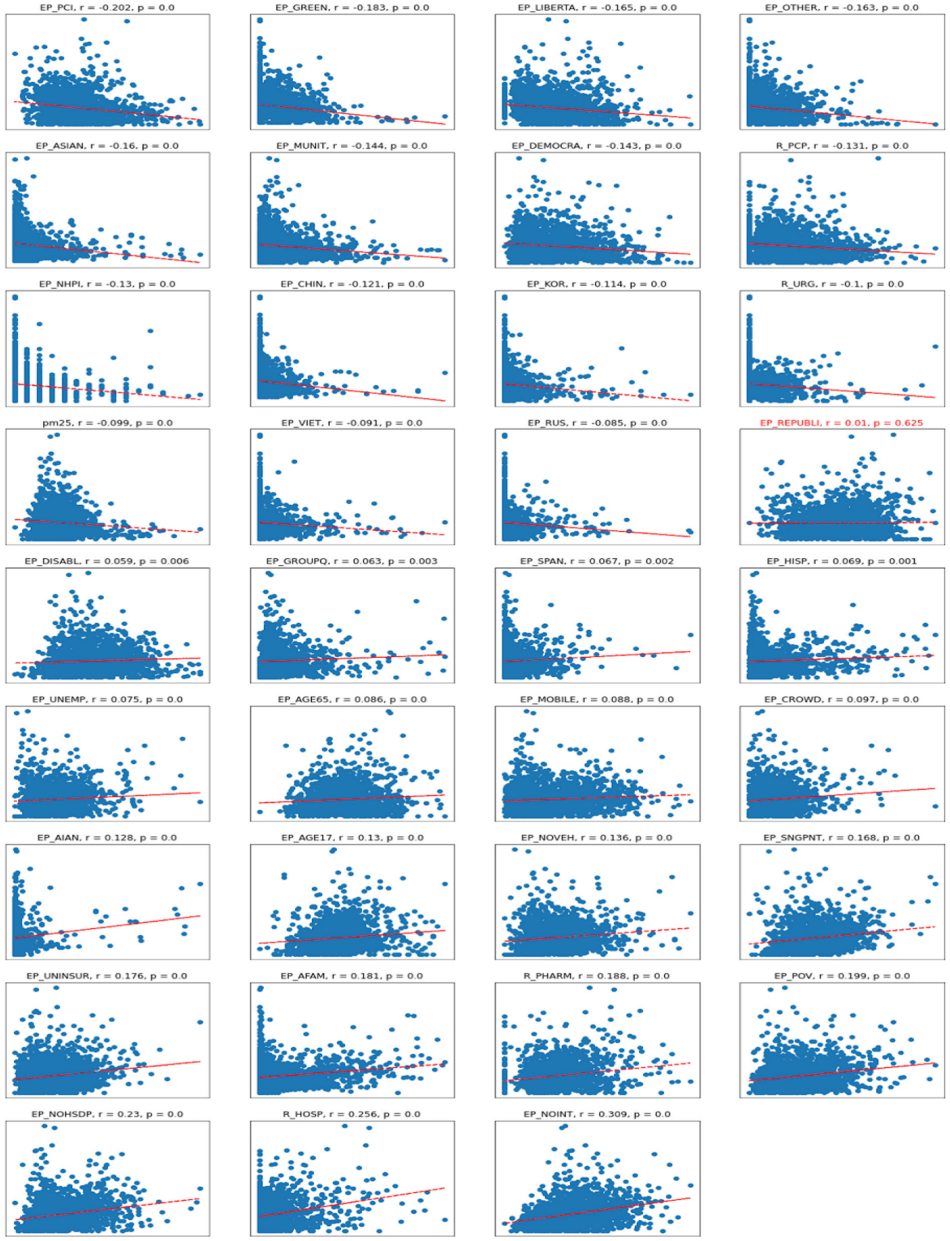
Scatterplots for Deaths vs. independent variables. *r* – Pearson correlation; *p* – significance.

Regarding the correlations between the number of deaths per 1,00,000 persons and various socioeconomic variables—the correlations are even milder than with the number of cases, meaning the deaths are even more difficult to predict reliably (Figure 5). The sets of variables with positive and negative coefficients are mostly the same but just ordered differently due to different correlation coefficients. The biggest negative correlation coefficient is with the per capita income; the lower the income is, the bigger the associated number of deaths. There are some mild negative associations with non-republican voters, and being of an Asian minority is associated with a lower number of deaths.

The biggest positive correlation is with lack of internet access, which could be associated with living in extreme poverty or remote places with no access to medical care. There is also a positive correlation with the number of hospitals, which could imply that a lot of those sick with COVID-19 died in hospital.

As one can see, there is a mild negative correlation between the dependent variable and the PM_2.5_ variable, and we will see similar behavior when examining regression coefficients. This could mean that the counties exhibiting the relatively high PM_2.5_ air pollution had relatively low case rates. If we look over the map, the air pollution is concentrated over the forested counties of Washington, Oregon, and California, likely due to the violent forest fires caused by dry weather that year. Because that air pollution was interpolated for the 10 months of 2020, such a model does not represent multiyear pollution concentrations. These long-term concentrations are perhaps more useful for the understanding of the developing underlying health conditions that could adversely affect cases and death rates. So the point here is that air pollution may or may not affect COVID-19 rates, but it is hard to draw any conclusions from the available data due to the short period of observations and the dynamic nature of the pandemic.

The next step of the analysis of the relationships between socioeconomic variables and COVID outcomes was to use an Ordinary Least Squares regression to look at regression coefficients to assess the predictive importance of these features. As a first step, variance inflation factors were printed to address multicollinearity issues in the dataset. The variance inflation factors are provided in Table 5.

**Table 5:** Variance inflation factors (VIFs) for different variables.

**Variable**	**VIF**	**Variable**	**VIF**
EP_OTHER	1.203574	EP_CHIN	2.629058
R_PHARM	1.421413	EP_CROWD	2.720431
EP_RUS	1.428143	EP_MUNIT	3.015496
R_HOSP	1.527417	EP_DISABL	3.284592
EP_GREEN	1.532433	EP_AFAM	3.587627
R_PCP	1.56123	EP_NOINT	4.294979
EP_KOR	1.643587	EP_AGE65	4.322232
EP_VIET	1.659622	EP_AGE17	4.329624
EP_NHPI	1.731772	EP_POV	4.702413
EP_LIBERTARIAN	1.85339	EP_PCI	4.850892
EP_GROUPQ	2.075243	EP_DEMOCRAT	4.861653
EP_AIAN	2.110417	**EP_ASIAN**	5.102229
EP_UNINSUR	2.282732	**EP_REPUBLICAN**	5.183313
EP_UNEMP	2.367726	**EP_NOHSDP**	5.460101
EP_SNGPNT	2.404438	**EP_HISP**	5.786559
EP_MOBILE	2.510499	**EP_SPAN**	6.617553
EP_NOVEH	2.544724		

Variance inflation factors (VIFs) > 5 are highlighted in bold.

The 'EP_SPAN', 'EP_REPUBLICAN', 'EP_ASIAN', and ‘EP_NOHSDP' variables were removed to make sure that there are no variables with VIFs equal to or >5. Having removed all these variables, the ordinary least squares regression was fit. There were two regression models fitted, one for the Number of cases per 1,00,000 persons as a dependent variable and the rest of the variables as independent, and another regression model for the number of deaths per 1,00,000 as a dependent variable, while other variables were treated as independent.

### 3.1 Cases

Fitting the OLS regression for case rates, the R2 for regression with all independent variables included was 0.41 and 0.405 with these variables removed. R2 metric for fivefold cross-validation was 0.39 with all variables and 0.38 without multicollinear variables. Also, the R2 metric was calculated for the unseen test data portion (25% of the data), and that metric was 0.37 with all variables and 0.35 without multicollinear variables. That means OLS regression can explain about 40% of the variability in the dataset.

**Figure 6:**
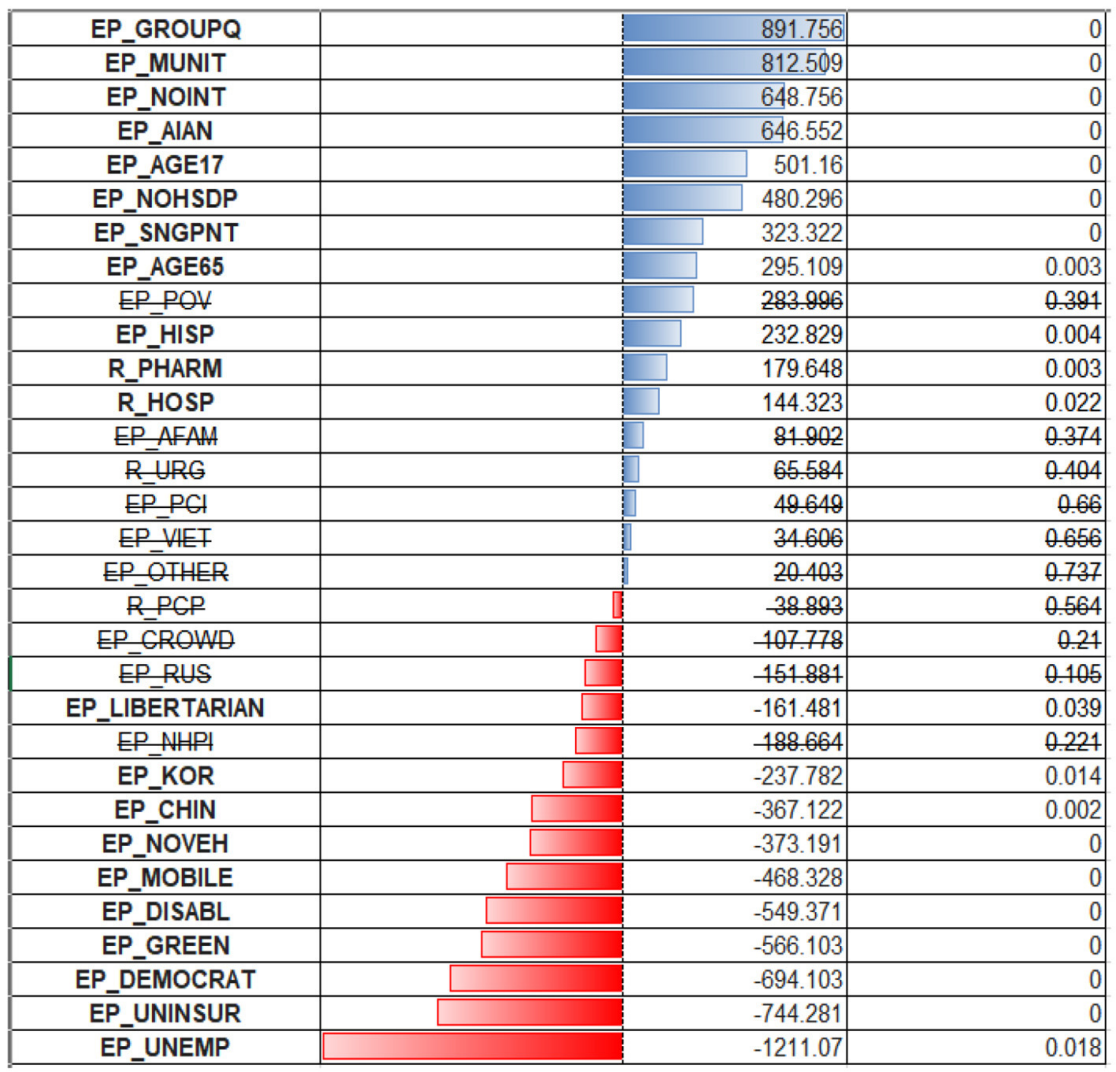
OLS regression coefficients for cases per 1,00,000.

From the inspection of the OLS regression coefficients (Figure 6), one can see that the model puts the biggest positive weight on the percentage of the population in group quarters (EP_GROUPQ variable), multiunit housing (EP_MUNIT), no access to the internet (EP_MUNIT), and being American Indian or Pacific Islander (EP_AIAN). The first two variables could indicate that living in crowded conditions and being financially disadvantaged could be associated with the increased spread of respiratory infection. All these considerations were true when we examined the correlation coefficients. What stands out is that being of age 65+ was previously associated with a negative correlation with cases ratio, but OLS regression puts a positive coefficient for this predictor (EP_AGE65). Variables depicting racial minority status (EP_AFAM, EP_VIET, EP_RUS, EP_OTHER) were deemed non-significant.

The largest negative coefficients were assigned to an unemployment rate (EP_UNEMP, EP_UNINSUR), lack of medical insurance, and political affiliation. The high weight assigned to the first two variables could be a coincidence of low testing rate and inaccessibility of health care for those without health insurance, so the cases are being significantly underreported due to reluctance/inaccessibility for these populations to be tested. There are also negative coefficients for the Chinese and Korean (EP_CHIN, EP_KOR) populations. One can speculate that there might be some association with specific healthy habits/improved sociodemographic status of these people.

### 3.2 Deaths

The ordinary least squares regression was similarly fitted for the death rates as a dependent variable. The R2 coefficient of determination was much smaller, which could indicate that the available set of predictors is less suited for the prediction of death rates than case rates.

R2 for regression with multicollinear variables included was 0.268, and 0.261 with these variables excluded. R2 for fivefold cross-validation was 0.229 for the dataset with all variables included, and 0.221 with multicollinear variables excluded. R2 for unseen test data (25%) dropped from 0.30 to 0.27 when multicollinear variables were excluded.

**Figure 7:**
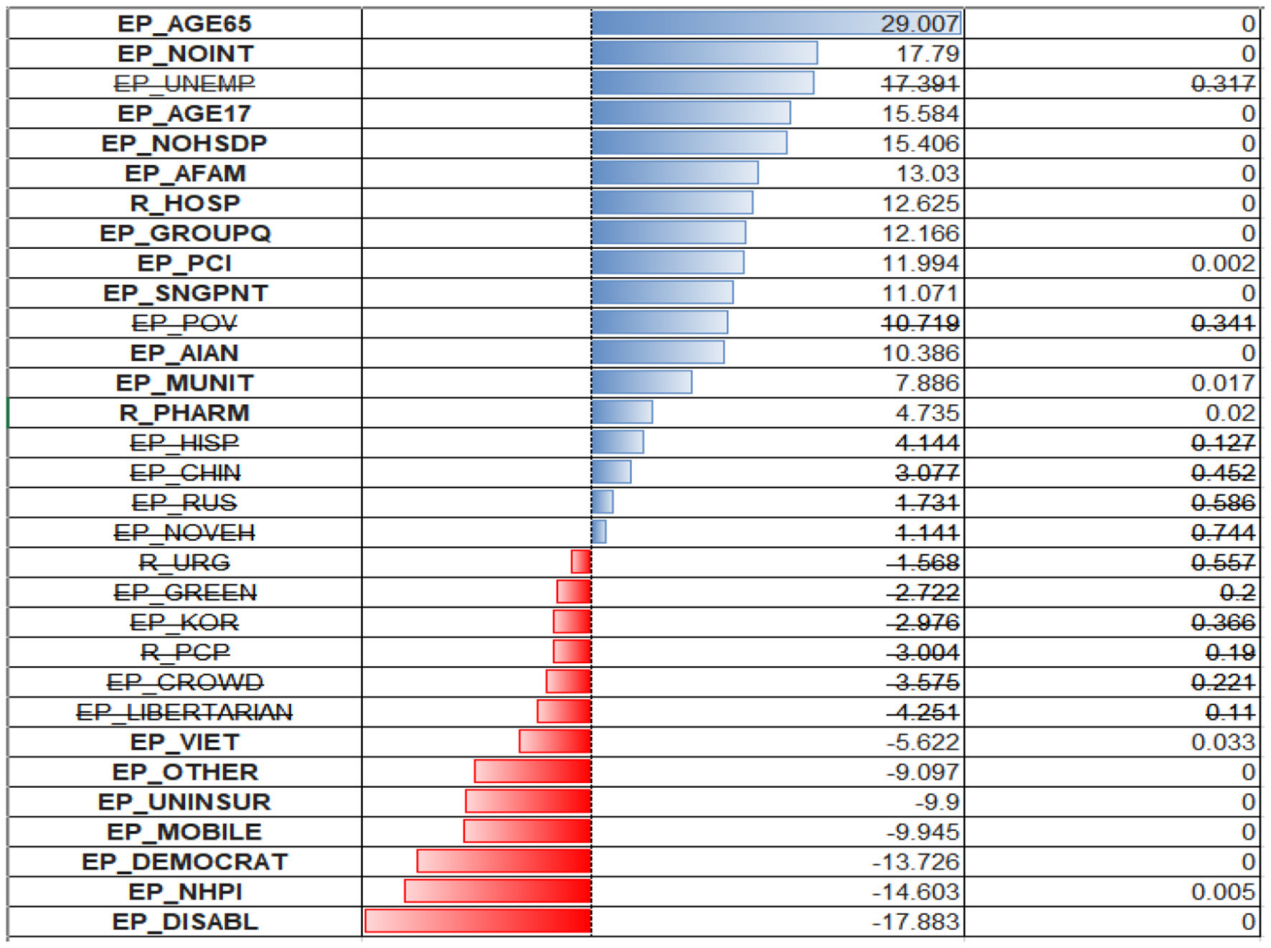
OLS regression coefficients for deaths per 1,00,000.

The model put the heaviest weight on the older adult variable (EP_AGE65). Other variables have lower weights, but their composition is like those of predictive value for case ratios. Surprisingly, the model assigns negative regression coefficients to the uninsured (EP_UNINSUR) and mobile homes population (EP_MOBILE) (Figure 7).

**Figure 8:**
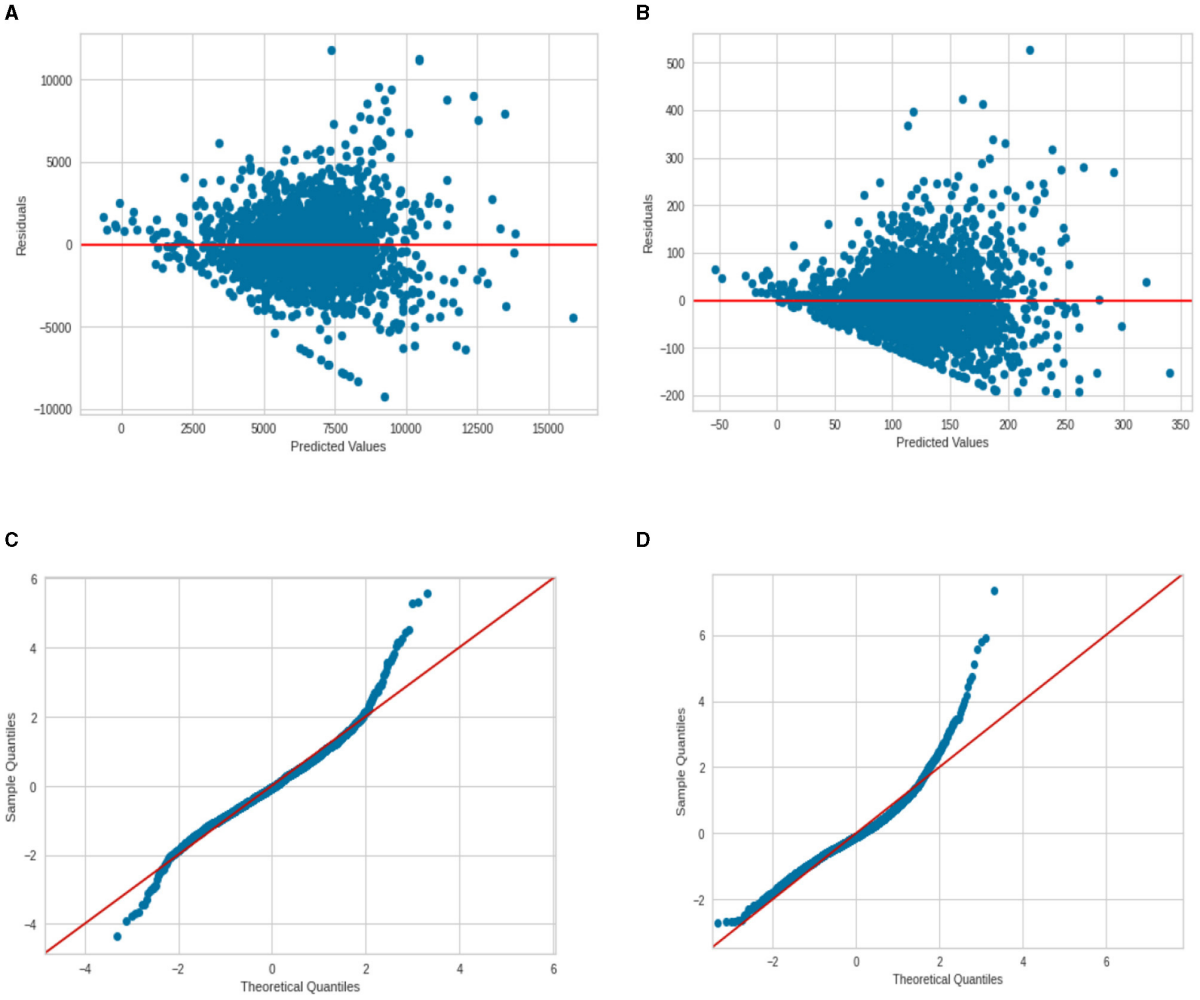
Regression diagnostic plots for cases and deaths per 100 000 **(A)** Residuals vs. Fit for cases; **(B)** Residuals vs. Fit for deaths; **(C)** normal Q–Q plot for cases; and **(D)** normal Q–Q plot for deaths.

One can find the OLS diagnostic plots for the two regression models in Figure 8. There is an indication of the non-constant variance for the residuals (higher predicted value associated with a larger prediction error). Also, the normal Q–Q plot shows evidence of a non-normal distribution of residuals—the points do not follow a straight line. This is more severe for the deaths per 1,00,000 persons model and less severe for the model cases per 1,00,000 persons.

As an additional step in regression analysis, a LASSO regression was employed. The search for the best alpha hyperparameter was performed using the LassoCV SKLearn module. The best alpha value was found as 12.984, which leads to the elimination of variables EP_AFAM (percentage of African American), EP_PCI (Per-capita income), EP_UNEMP (percentage of unemployed), EP_VIET (percentage of Vietnamese minorities), R_PCP (ratio of primary care providers per 100 000), and R_URG (ratio of urgent care providers). LASSO regression is important as it performs both variable selection and regularization, improving the prediction accuracy and interpretability of the resulting model. It is particularly useful for models that have high dimensionality or multicollinearity. In this case, the LASSO regression effectively reduced the complexity of the model by eliminating less-significant variables. A cross-validation R2 score experiences a very small drop compared to the OLS regression model.

### 3.3 Relationships between the spatial location and predicted values

To check if there are some systematic over/underpredictions that are geographically clustered, the regression residuals were mapped, and the Moran I test was performed for the residuals. The test was significant, with a *p*-value of 0 and a *z*-score of 61.05, which indicates a significant clustering of the residuals. This could mean that either there are some important predictors explaining such variability, for instance, information about the anti-COVID governmental policies, missing from the model, or there is a spatial process affecting the spatial distribution of cases/deaths ratios. Perhaps there are both; some variables are missing and travel patterns, closeness to the airports, and other spatial factors are affecting the spread of the disease.

To see, if the prediction accuracy improves, the RandomForestRegressor was used to predict cases/death ratios using the spatial coordinates of county centroids together with all other socioeconomic variables. There is a somewhat significant improvement in the prediction accuracy. Where one uses all the prediction variables [including multicollinear, as we only care about prediction here, not about how to interpret the model (inference)] with OLS, the R2 score was about 0.41 on the training data and about 0.37 using cross-validation. Contrary to that, if using an RF regressor with 100 trees, the cross-validation accuracy increased to 0.61 (about a 30% improvement).

**Figure 9:**
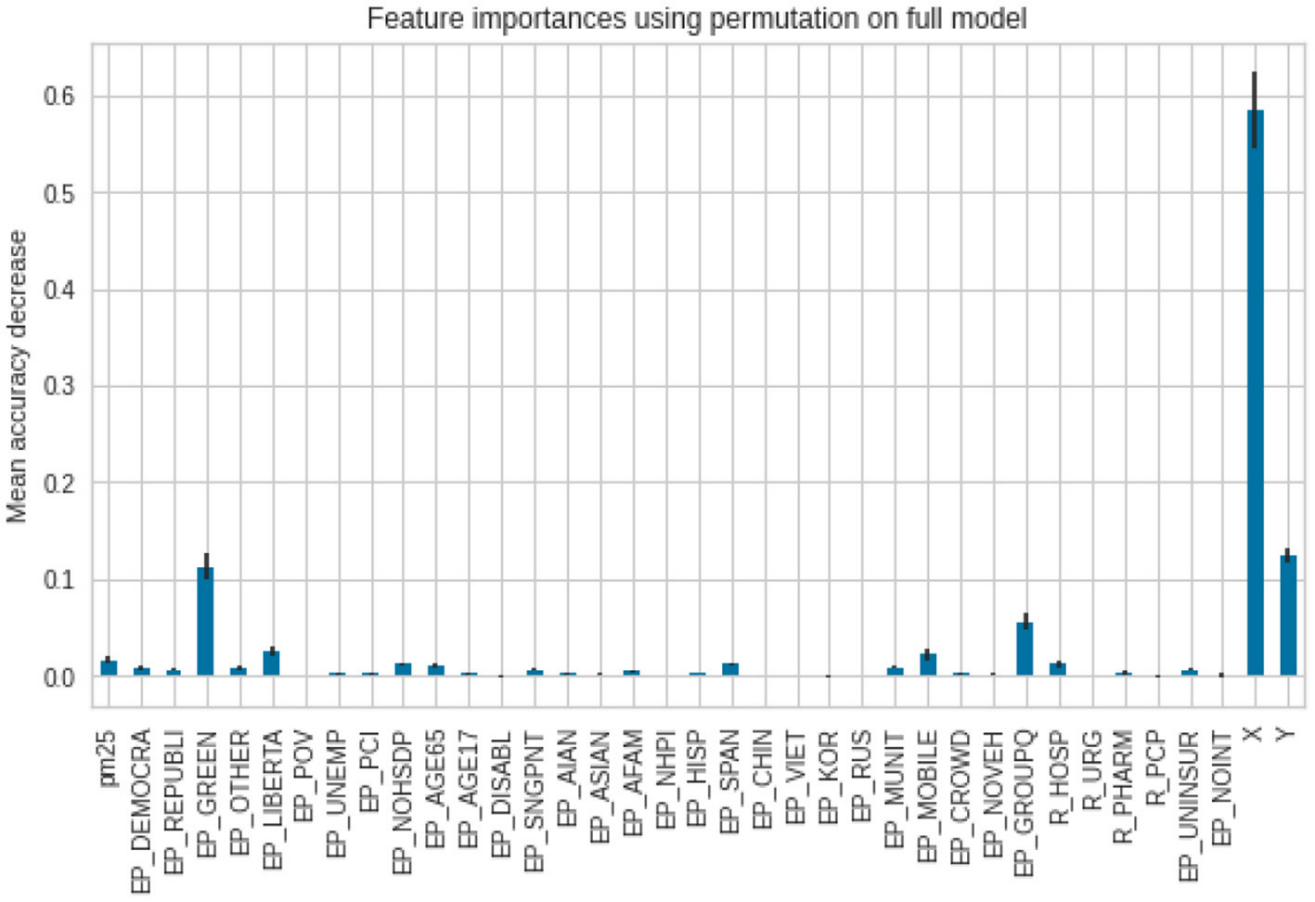
Feature importance scores for the cases per 1,00,000.

To assess feature importance, permutation importance scores were calculated and plotted. Regarding predicting the case ratios, as one can see, the model puts the biggest weight on the *X* and *Y* coordinates. The smaller weight was put on the affiliation with a Green party as well as with the population in group quarters. The importance of other variables is negligible (Figure 9).

**Figure 10:**
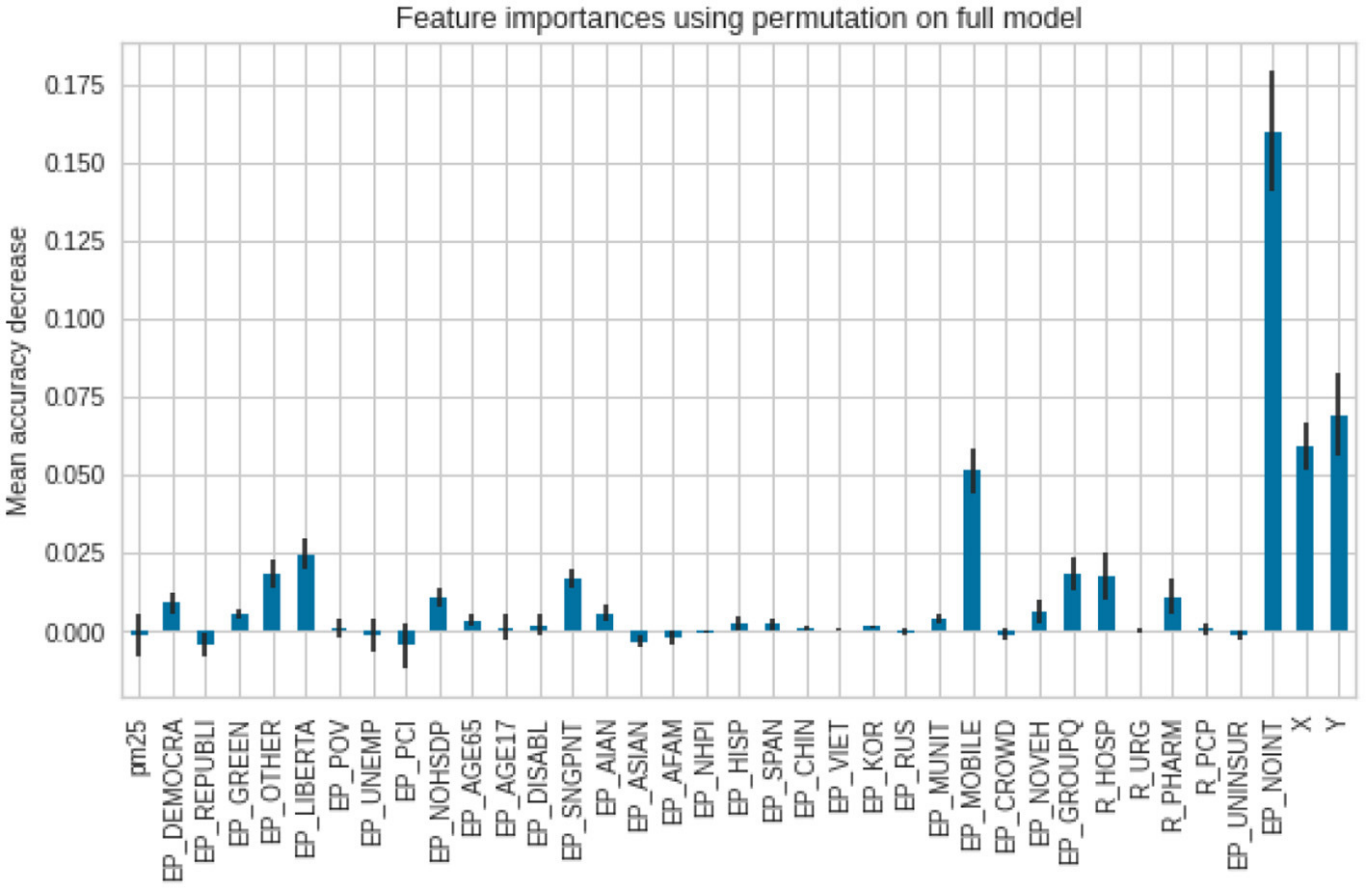
Feature importance scores for the deaths per 1,00,000.

As for the death ratios, the lack of internet access (EP_NOINT) was reported as the most important feature, then the spatial X and Y coordinates, followed by a percentage of the population, living in mobile homes (EP_MOBILE). The error bars in the second model (for deaths) are longer, which indicates reduced agreement between the different trees in the forest (Figure 10).

As an experiment, *X* and *Y* coordinates alone were used in a random forest regressor. It gave an R2 score of 0.45 for the unseen test data/0.49, using cross-validation for the cases per 1,00,000 persons. Regarding the prediction of deaths per 1,00,000, R2 is 0.23 on test data, and 0.28 is the mean R2 cross-validation score. The resulting accuracy scores were summarized in Table 6.

**Table 6:** Prediction accuracy comparison for different regression methods.

	**OLS without high VIF**		**Lasso**		**Random forest**		**RF for co-ords**	
	**Cases**	**Deaths**	**Cases**	**Deaths**	**Cases**	**Deaths**	**Cases**	**Deaths**
R2 training	0.405	0.286	0.402	0.285	0.949	0.909	0.933	0.905
R2 testing	0.349	0.242	0.348	0.220	0.599	0.313	0.455	0.239
5-fold CV sc	0.374	0.219	0.375	0.245	0.615	0.318	0.495	0.283

As one can see from the comparison of the R2 scores, the Random Forest regression shows the best results in predicting cases and death ratios. The second best-performing regression model is OLS with variables with high VIF removed. The LASSO model for cases performed very well; it removed six variables, creating a more parsimonious model, and the R2 scores still were almost the same as for the OLS model.

### 3.4 Relationship between COVID-19 and PM_2.5_ air pollution

The observed negative correlation in the scatterplots and regression models between cases/deaths per 1,00,000 persons and the PM_2.5_ ratio variable raises intriguing questions regarding the relationship between air pollution and respiratory health outcomes. While existing literature typically associates high PM_2.5_ concentrations with an elevated risk of respiratory issues, our findings suggest a counterintuitive scenario. It is crucial to consider the temporal aspects of exposure, as our study focused on a relatively short nine-month period for averaging and interpolating air pollution concentrations. This limited timeframe may not capture the nuanced and potentially delayed effects of prolonged exposure, leading to the development of underlying health conditions that could impact case and fatality ratios.

Moreover, the unique case of Western states like Washington, Oregon, and California introduces additional complexity to our interpretation. Despite facing significant forest fires, these states managed the pandemic relatively well and exhibited low case and death ratios per 1,00,000 persons. The occurrence of forest fires contributed to a temporary spike in PM_2.5_ air pollution, challenging conventional expectations. This transient elevation in air pollution may have created a confounding factor, resulting in the observed negative correlation. Thus, it is imperative to delve deeper into the dynamics of short-term vs. long-term exposure effects and consider regional variations in pollution sources and management strategies when elucidating the intricate interplay between air quality and public health outcomes.

The primary limitations of the study are the timeframe of the analysis, errors accompanied by considering select confounding factors, and limited resolution of air quality data.

## 4 Conclusions

In this research article, we studied the impact of sociodemographic characteristics, political affiliation, and air pollution on the variability in COVID-19 cases and fatalities per 1,00,000 people. According to the Ordinary Least Squares (OLS) regression model, ignoring spatial coordinates, these factors can account for ~40% of the variability in cases and 28% in deaths. Notably, greater regression coefficients indicate that age, population density, and wealth have significant effects. Minority status factors, on the other hand, do not achieve statistical significance at the 0.05 level. Adding spatial coordinates to a Random Forest (RF) regressor model improved prediction accuracy for cases by approximately 20%, but the increase in predicting deaths remained modest even with spatial coordinates. Surprisingly, utilizing the RF regressor with only location information explains around 45% of the variability in instances. However, including location and other variables only boosts explanatory power to 60%, indicating potential model inadequacies. Through this article, we also raised the concern of missing critical factors and emphasized the necessity for additional refining. Furthermore, the study's investigation into the association between PM_2.5_ air pollution and health outcomes yielded inconclusive results, prompting the call for more solid, long-term estimates from sources such as the government (AirNow.gov) network to improve accuracy in future research. This article reinforces the fact that one should be mindful and prudent about making any affirmative causal statements regarding air quality constituents (e.g., PM_2_._5_) and socioeconomic factors contributing toward COVID-19 morbidity and/or mortality rates as age and health status are primary factors, governing the extent of adverse impacts people experience due to COVID-19. Within this context, this study further reinforces the need for the implementation of better health practices (e.g., consistent exercise routine, healthy eating/appropriate nutrient intake, sleeping enough, no smoking, etc.), that mitigate underlying preconditions (e.g., cardiovascular disease, hypertension, diabetes, chronic obstructive pulmonary disease, and severe asthma, kidney failure, severe liver disease, immunodeficiency, and malignancy) that amplify the adverse impacts of COVID-19.

## Data availability statement

The original contributions presented in the study are included in the article/supplementary material, further inquiries can be directed to the corresponding author.

## Author contributions

NG: Conceptualization, Data curation, Software, Validation, Visualization, Writing – original draft, Writing – review & editing, Formal Analysis, Investigation, Methodology. SW: Formal analysis, Validation, Visualization, Writing – original draft, Writing – review & editing. MY: Conceptualization, Project administration, Software, Visualization, Writing – original draft, Writing – review & editing. NK: Conceptualization, Formal Analysis, Investigation, Software, Validation, Writing – original draft, Writing – review & editing. OI: Formal analysis, Validation, Writing – original draft, Writing – review & editing. BA-H: Methodology, Software, Validation, Writing – original draft, Writing – review & editing. CB-B: Conceptualization, Data curation, Project administration, Software, Validation, Visualization, Writing – original draft, Writing – review & editing.
